# Supplementary material for: Correction: Secure deep learning for distributed data against malicious central server
Source: PLoS One. 2025 Jan 23;20(1):e0318164. doi: 10.1371/journal.pone.0318164 (PMC11756750; doi:10.1371/journal.pone.0318164)
Supplement: S2 File — (PDF) [file pone.0318164.s002.pdf]

PLOS ONE

RESEARCH ARTICLE

Secure deep learning for distributed data against malicious central server

Le Trieu Phong

National Institute of Information and Communications Technology (NICT), Koganei, Tokyo, Japan

* phong@nict.go.jp

Abstract

In this paper, we propose a secure system for performing deep learning with distributed trainers connected to a central parameter server. Our system has the following two distinct features: (1) the distributed trainers can detect malicious activities in the server; (2) the distributed trainers can perform both vertical and horizontal neural network training. In the experiments, we apply our system to medical data including magnetic resonance and X-ray images and obtain approximate or even better area-under-the-curve scores when compared to the existing scores.

Check for updates

OPEN ACCESS

Citation: Phong LT (2022) Secure deep learning for distributed data against malicious central server. PLoS ONE 17(8): e0272423. https://doi.org/10.1371/journal.pone.0272423

Editor: Hua Wang, Victoria University, AUSTRALIA

Received: February 17, 2022

Accepted: July 19, 2022

Published: August 1, 2022

Copyright: © 2022 Le Trieu Phong. This is an open access article distributed under the terms of the Creative Commons Attribution License, which permits unrestricted use, distribution, and reproduction in any medium, provided the original author and source are credited.

Data Availability Statement: All relevant data are within the article.

Funding: This work is partially supported by JST CREST, Japan, Grant JPMJCR21M1. The funders had no role in study design, data collection and analysis, decision to publish, or preparation of the manuscript.

Competing interests: NO authors have competing interests.

1 Introduction

Deep learning is a set of machine learning techniques that has gained much interest in recent years, owing to its potential applications in many fields. In the field of medicine, deep learning applications such as the classification of skin cancer [1], detection of diabetic retinopathy [2], and detection of pneumonia [3] and COVID-19 [4] using chest X-rays, have recently shown considerable potential to improve the quality of healthcare for patients worldwide.

Medical data are by nature vastly distributed, as they often originate from several hospitals and medical centers throughout the world. It is reported in [5] that thousands of exabytes (10^{18} bytes) of medical data will be generated. To accelerate the availability and accuracy of deep learning in healthcare through the use of this kind of big data, deep learning systems using distributed data should be designed and examined. However, because medical data are personal and sensitive, serious attention should be paid to securely protect these data.

Pioneering efforts for designing deep learning systems with distributed data have been in the works of Recht et al. [6] and Dean et al. [7], whose systems are then considered and expanded in the security and privacy domain by Shokri and Shmatikov [8]. All of these works assume a central parameter server that is responsible for updating the parameters of the neural network model in the deep learning systems. Specifically, in [6, 7], the server is assumed to be completely honest in operation, whereas in [8] the server is semi-honest (i.e. honest-but-curious) which signifies that it is honest in operation but curious in extracting sensitive information. Subsequent works with security improvements include [9, 10] which also assume a semi-honest parameter server. The systems of federated learning in [11, 12] have been experimentally demonstrated to be able to handle non independent and identically distributed (non-iid)

Table 1. Comparison of area-under-the-curve (AUC) scores.

Learning utility AUC score (→) Dataset (↓)	Our system (securely distributed)	Best known (centralized)
MRI (Stanford, Croatia)	0.924	0.911
X-ray (ChestX-ray14)	0.839	0.841

https://doi.org/10.1371/journal.pone.0272423.t001

data experimentally; however the systems are not for vertical training due to the average calculation of neural network weights over the central parameter server.

1.1 Our contributions

In this work, we propose a secure system for deep learning using distributed datasets. Our system has the following features:

- **Detection of malicious activities in the parameter server**: Our system is designed so that a malicious parameter server is detected with overwhelming probability. This is made possible by a novel use of authenticated encryption, in which the encryption part protects communication secrecy whereas the authentication part detects any changes in the communication.
- **Both vertical and horizontal training**: Our system can handle both vertical and horizontal training by design. For vertical training, we mean a training model produced by one trainer on a dataset can be re-used by another trainer on an entirely different dataset after proper modifications. For horizontal training, we mean a shared model is trained in a distributed manner using local datasets of the trainers. It is also worth noting that, besides improving the utility of the system, the combination of vertical and horizontal model training can produce robustness with respect to noisy labels as discussed in Section 3.3.
- **Experimentation with distributed medical data**: On chest X-ray images [13] and magnetic resonance imaging (MRI) images [14, 15], we demonstrate that our securely distributed system either approximates or outperforms existing results in the literature in which the data had to be centralized. Indeed, as showed in Table 1, the learning utility scores of our system in terms of area-under-the-curve (AUC) are very close to (or better than) the best known scores in non-distributed (centralized) training, as seen in Table 1. More detailed comparisons of AUC scores are given in Tables 3 and 4, again showing that the AUC scores of our system are very similar and in some cases superior to the best known scores.

1.2 Related works

All existing systems in [8–12] cannot detect malicious activity of the parameter server due to the fact that plaintexts or malleable ciphertexts are directly handled by the server. For example, in [9], a homomorphic ciphertext $\mathsf{HEnc}(G)$ of a gradient vector G encrypted by a homomorphic encryption scheme HEnc is sent to the parameter server. If the server is malicious, it can modify that ciphertext by the following homomorphic calculation

$$\mathsf{HEnc}(G) + \mathsf{HEnc}(\epsilon) = \mathsf{HEnc}(G + \epsilon),$$

where ϵ is a vector intentionally selected by the server. In turn, the distributed trainers obtain $G + \epsilon$ instead of G without noticing, which is undesirable.

Likewise, the system in [10] cannot detect malicious activities in the server due to the use of symmetric malleable encryption such as the Cipher Block Chaining (CBC) mode with the Advanced Encryption Standard algorithm. Indeed, the encryption of a vector W in the first

block of the CBC mode by a symmetric key K is of the form

$$IV, \mathsf{AES}_K(IV \oplus W),$$

and the malicious server can modify that ciphertext into

$$IV \oplus r, \mathsf{AES}_K(IV \oplus W),$$

where r is selected by the server. The decryption of the modified ciphertext is

$$(IV \oplus W) \oplus (IV \oplus r) = W \oplus r,$$

which is obtained by a distributed trainer instead of W without any awareness. This subsequently affects other blocks in the decryption and the entire training process, and a distributed trainer cannot identify whether the malfunctions originate from the server or other trainers.

The proposed system in this paper extends [10] in the following directions of both security and learning utility: (1) we introduce authenticated encryption into the system to handle the malicious parameter server; (2) we make vertical training possible at each distributed trainer; (3) we perform experiments on medical imaging data to demonstrate the learning utility of the system in terms of AUC scores. However, it significantly deviates from [9] which is based on [7] (whose system is later restructured into TensorFlow [16].)

Techniques for differential privacy [17–21] or anonymous transmission [22] can be used locally at each distributed trainer in our system to protect the privacy or the origin of the transmitted weights. Likewise, each distributed trainer can deploy preventive measures such as in [23] if necessary. These techniques are useful for protecting the weight privacy to the greatest extent possible while maintaining the learning utility of the system. It is also worth noting that requiring the weight to contain no information on the data can be fulfilled if each trainer continues transmitting uniformly random weights; however this kind of "perfectly private" system has no learning utility at all. Requiring the neural network weight sent from an honest trainer to contain no information on the data, while maintaining the learning utility of the system, is impossible in the setting of collaborative training [24].

Model weight inversion attacks such as in [25, 26] have limited impacts and they do not necessarily entail a privacy breach as discussed in [27]. Similarly, the use of generative adversarial networks for attack on collaborative training systems [28] has been reported to be unrealistic in [29]. In addition, it is known in the literature that attacks on neural network weights are apparently more difficult than neural network gradients, on which various attacks and corresponding defenses exist (e.g. [9, 28, 30, 31]). In contrast, weights can be viewed as a large aggregation of gradients and are thus more resistant to attacks as observed in [10, 32].

Secure linear models have been studied in several works [33–38] with threat models ranging from semi-honest to malicious adversaries. For example, the system in Zheng et al. [34] utilizes threshold homomorphic encryption, zero knowledge proofs, and malicious multi-party computation to deal with malicious adversaries.

Aiming to achieve both secrecy and differential privacy, Aono et al. [39, 40] have designed systems for privacy-preserving linear and logistic regression, in which a semi-honest central server is used to handle homomorphic ciphertexts. Semantic security with homomorphism allows their system to achieve data secrecy (with respect to the central server) and differential privacy (with respect to publishing the final result) simultaneously. However, their technique of polynomial approximation of non-linear functions as in [40] appears to have limitations when applied to deep neural networks with multiple layers.

Using two non-colluding servers on the cloud, Mohassel and Zhang [41] have proposed protocols for privacy-preserving linear regression, logistic regression, and multilayer

perceptron in which secure-computation-friendly activation functions are employed. Subsequently, Mohassel and Rindal have also considered a three-server model in [42], in which data owners secretly share their data among three servers that train and evaluate models on the combined datasets using three-party computation.

Several works [43–48], especially in the framework of secure outsourced computation, have examined the problem of secure neural network prediction in which predicted probabilities for individual data items can be obtained in a secure manner. This vein of research on secure prediction is orthogonal to the topic in this paper which focuses on securely distributed training.

Chang et al. [49] have proposed a system for distributed deep learning and experimented with medical datasets, without a central parameter server. The system and the experiments are designed for horizontal training. Gupta and Raskar [50] have designed a method for distributed training where pieces of information such as data labels and neural network gradients are transmitted among distributed trainers. McClure et al. [51] have considered distributed training with a specific neural network only. These works have no explicit security considerations.

Various machine learning algorithms involving multiple parties can be securely operated over completely trusted hardware. However, even in such setting, care should still be taken to guard the algorithms from memory access patterns that depend on data, as examined in [52]. Techniques for federated learning (e.g., [12, 53]) and subsequent works (e.g., [54–58]) can be used for distributed data, but they do not consider malicious central server as in our setting.

Generic secure multiparty computation (MPC) using secret sharing [59, 60] can securely compute any function represented as arithmetic circuits. The known weakness of such protocols is in the communication costs [11]. To address the issue, a dedicated protocol for secure aggregation in federated learning has been also proposed in [11]. In works such as [11] or subsequent [61], the server learns the full or partial sum of the trainers' inputs; which is orthogonal to our work in which the server cannot learn that kind of information. Works combining differential privacy with MPC (e.g., [62]), often admitting accuracy degradation due to noise addition, are also orthogonal to our work.

2 Preliminaries

We recall a few preliminaries on cryptography and machine learning in this section.

Authenticated encryption

Symmetric encryption schemes consist of the following (possibly probabilistic) polynomial-time algorithms: $\mathsf{KGen}(1^\kappa)$ takes a security parameter κ and generates secret key K; $\mathsf{Enc}(K, m)$, also written as $\mathsf{Enc}_K(m)$, produces c which is the ciphertext of message m; and $\mathsf{Dec}(K, c)$ or $\mathsf{Dec}_K(c)$ returns message m encrypted in c.

The security notion of ciphertext integrity (INT-CTXT) [63] requires that it be computationally infeasible to produce a ciphertext not previously produced by the holder of key K. In addition, ciphertext indistinguishability against chosen plaintext attacks (IND-CPA) ensures that no information is leaked from ciphertexts. Our system employs symmetric encryption with both ciphertext integrity and ciphertext indistinguishability.

A generic construction that achieves INT-CTXT and IND-CPA simultaneously is the composition of encrypt-then-mac, where mac refers to message authentication code. Namely, an authenticated encryption scheme can be constructed as follows

$$\mathsf{Enc}_{K_e \| K_a}(m) = C \| \mathsf{MAC}_{K_a}(C)$$

where $C = \mathsf{Enc}^{cpa}_{K_e}(m)$ where $\mathsf{Enc}^{cpa}_{K_e}(\cdot)$ is an encryption algorithm in an IND-CPA-secure

symmetric encryption scheme, and $\mathsf{MAC}_{K_a}(\cdot)$ is a message authentication code. The keys for encryption and authentication K_e and K_a must be independent and generated uniformly at random by the key generation algorithm $\mathsf{KGen}(1^\kappa)$. It has been proved in [63] that when the message authentication code is strongly unforgeable then the encrypt-then-mac composition satisfies both INT-CTXT and IND-CPA notions of security. It should be noted that a weaker notion of integrity called plaintext integrity in [63] can also be used if one only needs to determine whether the plaintext (i.e. neural network weight) inside the ciphertext has been modified. This weaker notion of integrity leads to broader compositions of cryptographic primitives for authenticated encryption that can be used in our proposed system.

Neural networks

In each distributed trainer is a neural network. The neuron (including the bias) nodes are connected via weight variables W, which can be considered a real vector. In a deep learning neural network structure, there can be multiple layers each containing thousands of neurons. Each neuron node (except for the bias node) is associated with an *activation function* f. Typical examples of f can be $f(x) = \max\{0, x\}$ (rectified linear), and $f(x) = \frac{e^x}{e^x+1}$ (sigmoid). The nonlinearity of these activation functions is important for the network to learn complex data distributions.

Given a training dataset, the learning task is to determine these weight variables to minimize a predefined cost function such as the cross-entropy cost function detailed later in each experiment in Section 4.

3 Our system

3.1 System description

The proposed system makes use of an authenticated encryption scheme ($\mathsf{KGen}(1^\kappa)$, $\mathsf{Enc}_K(\cdot)$, $\mathsf{Dec}_K(\cdot)$) with ciphertext integrity [63], where κ is a security parameter and K is a symmetric key generated by the key generation algorithm $\mathsf{KGen}(1^\kappa)$. A figurative and algorithmic illustration is provided in Fig 1. The system follows and generalizes the system in [10] in the following ways: (1) we introduce authenticated encryption into the system to address a malicious parameter server; (2) we insert vertical training into the distributed trainers so that they can handle more types of datasets.

Below, $M \circ M_i$ denotes a modification of model M by distributed trainer i. Specifically, if M has a inputs and b outputs, and M_i has b inputs and c outputs, then $M \circ M_i$ is the composed model of a inputs and c outputs. Mathematically, if $M : \mathbb{R}^a \to \mathbb{R}^b$ and $M_i : \mathbb{R}^b \to \mathbb{R}^c$ then $M \circ M_i : \mathbb{R}^a \to \mathbb{R}^c$ as the composed model. A visualization is given in the neural network model of trainers in Fig 1.

The proposed system:

- **Initialization (for all distributed trainers):** common model and cryptographic key setup. This can be done by Trainer 0 in the system.
  - Generate a common neural network model M.
  - Generate a cryptographically symmetric key K using $\mathsf{KGen}(1^\kappa)$.
  - Share model M and key K to all distributed trainers (but not the parameter server) via a secure channel.
- **Central parameter server:**
  - When receiving ciphertext E from a distributed trainer, store it.

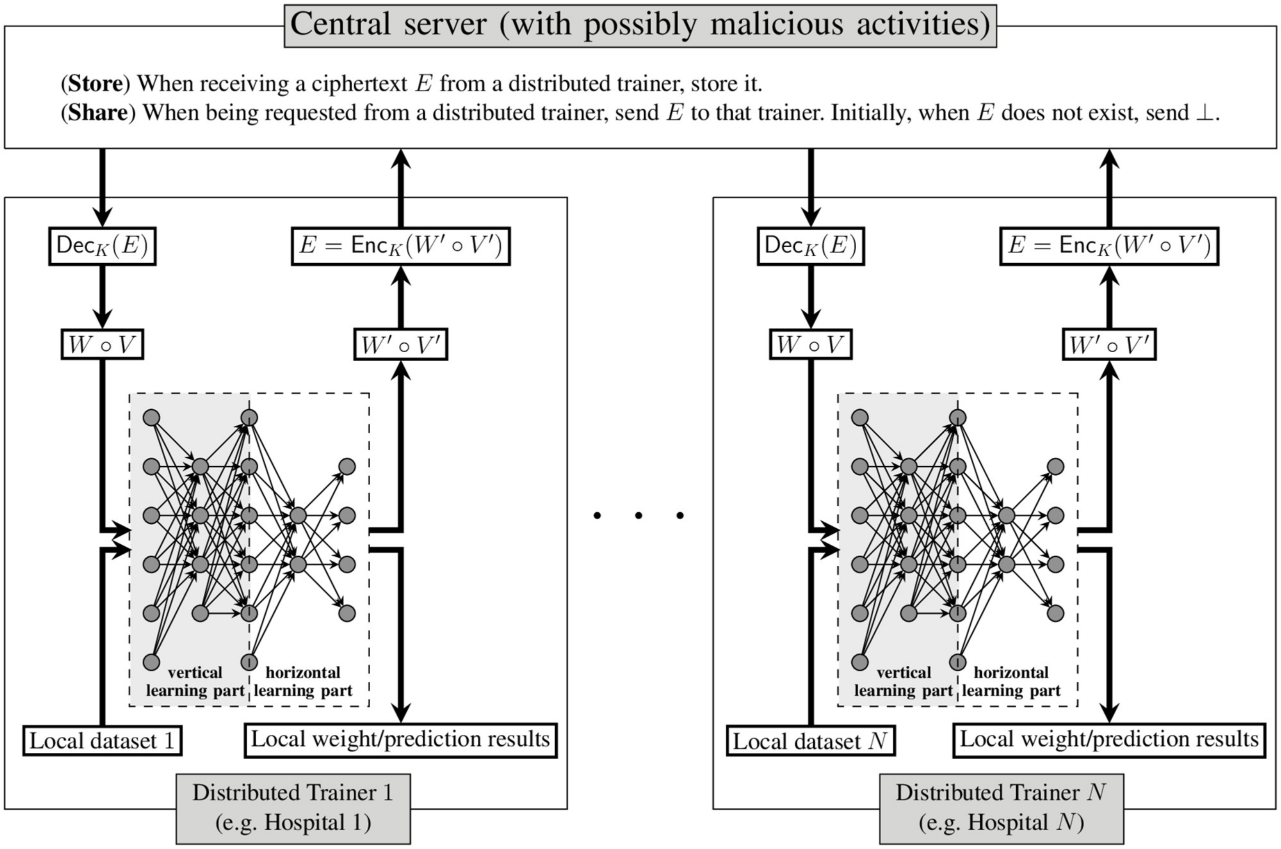

Fig 1. Our system of deep learning for both horizontal and vertical training that can detect malicious activities in the server.

https://doi.org/10.1371/journal.pone.0272423.g001

- When receiving a request from a distributed trainer, send E to that trainer. It is also possible that the server decides which trainer to send E. Initially, when E does not exist, send $\perp$. If E has been sent, wait for the encrypted post-trained weight from the requested trainer.

- **Each distributed trainer *i*:**
  - Generate neural network model $M \circ M_i$ where M_i is a model generated by trainer i.
  - Obtain encrypted weight E from the central parameter server. If $E = \perp$, initialize the weight for $M \circ M_i$. If $E \neq \perp$, decrypt E to obtain the pre-trained weight $W \circ V$, namely $W \circ V = \mathsf{Dec}_K(E)$.
  - Starting from $W \circ V$, train the neural network model $M \circ M_i$ using the local data of trainer i to obtain the post-trained weight $W' \circ V'$. Set $W \circ V \leftarrow W' \circ V'$.
  - Send the encrypted $E = \mathsf{Enc}_K(W \circ V)$ to the central parameter server.

Particular usage of our system: Vertical then horizontal training. The initial trainer (e.g., Trainer 0) queries the server and obtains $E = \perp$, so it proceeds to train $M \circ M_0$ where M_0 is null so that the model $M \circ M_0 = M$, namely the model is unchanged. Therefore Trainer 0 initializes weight W and trains model M with W on its data to obtain the post-trained weight W'. Trainer 0 sets $W \leftarrow W'$ and sends $E = \mathsf{Enc}_K(W)$ to the central parameter server.

The next trainer (e.g. Trainer 1) queries the server and gets $E = \mathsf{Enc}_K(W)$. It then decrypts the ciphertext and obtains weight W as the pre-trained weight for model M. Trainer 1

generates M_1 and composes $M \circ M_1$. It then initializes V and feeds $W \circ V$ into $M \circ M_1$. Starting from $W \circ V$, Trainer 1 trains model $M \circ M_1$ using its local data to obtain $W' \circ V'$. It then sets $W \circ V \leftarrow W' \circ V'$ and sends $E = \mathsf{Enc}_K(W \circ V)$ to the central parameter server so that other trainers can continue the training process. Other trainers (Trainers 2, . . ., N) behave similarly to Trainer 1.

3.2 Security considerations for our system

Our system has a stronger security guarantee against the central parameter server than previous systems in [9, 10]. Details are provided below.

Detecting a malicious server by any trainer. By a malicious server, we mean a server interested in extracting information about the data of the trainers. To accomplish that goal of information extraction, the server may even try to modify the incoming ciphertext before sending it to another trainer. In our system, if the central parameter server maliciously modifies ciphertexts uploaded by the trainers, the trainers can detect the malicious activity.

This is by design; because the ciphertexts have integrity, thus it is computationally infeasible to produce a ciphertext not previously produced by the trainers. Specifically, if the mode of encrypt-then-mac [63] is used, then the message authentication code (e.g. HMAC [64]) can detect whether a ciphertext has been changed or not. Let $K = (K_e, K_a)$ consist of the keys for symmetric encryption K_e and message authentication code K_a. As described in Section 2,

$$\mathsf{Enc}_{K_e \| K_a}(W \circ V) = C \| \mathsf{MAC}_{K_a}(C)$$

for the weight vector $W \circ V$. As a result, any change to C and weight vector $W \circ V$ can be detected by the distributed trainers with the common authentication key K_a of the MAC.

Security for a trainer against malicious trainers and server, and their collusion. This scenario is identical to that in [10] black (Section IV); thus our proposed system inherits the security results in [10]. In particular, our system ensures security in terms of onewayness for any honest trainer to the greatest extent possible. As mentioned in Section 1.2, requiring that the neural network weight sent from an honest trainer possesses no information on the data, while maintaining the learning utility of the system, is infeasible in the setting of collabo-rative training [24]. Regarding this point, various defenses have been discussed in [10], including the use of differential privacy (e.g. [20]), anonymous transmission (e.g. [22]), and adversarial regularization [23] to protect the weight (and its origin) of the honest trainer. The honest trainer does not send individual gradients of small batch sizes; thus it can resist attacks on gradients such as in [9, 28, 30, 31].

It is also worth remarking that, if a malicious trainer injects noise into the training process, then the noise can also be manually detected by an honest trainer by locally observing training indicators such as training loss and AUC scores.

Nonetheless, it should be noted that our system is in the cross-silo scenario, in which trainers are large organizations such as medical or financial institutions with certain responsibilities required by regulations. Therefore, we expect that the case of malicious trainers (and server), and their collusion, is less likely to happen than the case of a malicious server alone.

3.3 Learning utility robustness via vertical training

The vertical training of Trainer 0 on a dataset with clean labels can improve model robustness against noisy labels of subsequent trainers. This is because deep neural networks have the ability to memorize patterns in the initial epochs (i.e. the vertical training phase in our context) as observed in [65, 66]. This is particularly true in our experiments in which Trainer 0 employs the ImageNet (http://www.image-net.org/) dataset, and other trainers use medical datasets

that may have a portion (e.g. approximately 10% in the ChestX-ray14 dataset [13]) of inaccurate labels due to the process of automatic labeling from texts via natural language processing. To the best of our knowledge, this property of learning utility robustness has not been achieved in previous works [8–12] whose systems assume that labels are clean and accurate.

4 Experiments with medical data

All experiments employ a machine with Intel(R) Xeon(R) CPU E5-2699 v4 @ 2.20GHz and GPU NVIDIA P-100; with Python 3.7.2 distributed in Anaconda 4.5.11. We assume a standard 1 Gbps channel between the trainers and the server.

For authenticated encryption, the encrypt-then-mac method [63] is employed in which AES-256-CBC encryption is for the encryption part and HMAC-SHA512 (in OpenSSL 1.1.1a) is for the message authentication code part. Using more dedicated modes or hardware for authenticated encryption can improve the speed of encryption and decryption.

4.1 Experiment with MRI datasets

Trainers and datasets. In this experiment we suppose 3 distributed trainers:

- Vertical trainer 0 with the ImageNet dataset,
- Horizontal trainer 1 with an MRI dataset collected from Stanford University Medical Center [14], and
- Horizontal trainer 2 with an MRI dataset from Clinical Hospital Centre Rijeka (Croatia) [15].

The MRI dataset from Stanford contains 1130 exams, of which 208 exams have anterior cruciate ligament (ACL) tear, whereas the others (1130–208 = 922) do not. In addition, while each exam contains various types of images, only sagittal ones are compatible with the sagittal images from Croatia, and thus selected for distributed training in our system.

The Croatia training dataset contains 552 exams with sagittal series of images, of which 139 has label 1 and 413 has label 0. The Croatia validation dataset contains 38 exams of label 1 and 143 exams of label 0. The test dataset has 50 exams of label 1 and 134 exams of label 0. These distributions of labels are summarized in Table 2.

Neural network model. Following [14], we employ AlexNet [67] as the base neural network model in our system. Trainer 0 trains AlexNet using the ImageNet dataset. The trained weight from Trainer 0 is sent securely to Trainer 1 via the central parameter server. Trainer 1 and 2 modify M as follows: each MRI series of images $s \times 3 \times 224 \times 224$ is passed through a feature extractor based on AlexNet (= M) to obtain a $s \times 256 \times 6 \times 6$ tensor; a global average pooling layer and max pooling are then applied sequentially to reduce that tensor to $s \times 256$ tensor and 256 real numbers respectively; the last layer has one node fully connected with 256 nodes of the previous layer. These neural network models of Trainer 1 and 2, denoted as $M \circ M_1$ and $M \circ M_2$, contains 61,101,097 trainable parameters, having an approximate size of 234 MB when saved to disk.

Table 2. Label distribution in MRI datasets.

	Label 1 quantity	Label 0 quantity
Stanford dataset [14]	208	922
Croatia dataset [15]	139	413

https://doi.org/10.1371/journal.pone.0272423.t002

Authenticated encryption of model weights. We use encrypt-then-mac method which is proved to be authenticated encryption [63], whose running time is less than 3 seconds when applied on a model weight of size 234 MB. The ciphertext is also of 234 MB when saved to disk, and needs less than 3 seconds to be transmitted to the central parameter server. It is worth noting that the running times of encrypt-then-mac (3 seconds) and encrypted weight transmission (3 seconds) are relatively small when compared with the time for training (feed-forward and backpropagation on GPU), which are approximately 13 seconds for one epoch on the Croatia training dataset, and 39 seconds for one epoch on the Stanford training dataset.

Loss function for training. Trainers 1 and 2 use the same loss function of binary cross entropy with weights depending on the number of labels in the (joint) training set. More precisely, for a single data item (X, y) in the training set, the loss function is defined as

$$\begin{aligned} L(X,y) &= -w_{(1)} \cdot y \cdot \log \Pr[Y=1|X] \\ &\quad -w_{(0)} \cdot (1-y) \cdot \log \Pr[Y=0|X] \end{aligned}$$

in which

$$\begin{aligned} w_{(1)} &= \frac{139+208}{552+1130} \approx 0.2063 \\ w_{(0)} &= 1-w_{(1)} \approx 0.7937 \end{aligned}$$

because 139 (out of 552) and 208 (out of 1130) are the numbers of 1 in the Croatia and Stanford datasets respectively.

Training details. Trainer 1 and 2 follow the training procedure in [14] to optimize the above loss function for 20 central epochs. Each trainer executes at most two local epochs on its data before encrypting and sending the trained weight. For example the order of training can be: (1, 2, 2, 1, 1, 2, 1, 2, 2, 1, 1, 2, 1, 2, 1, 2, 2, 1, 1, 2, 1, 2, 1, 2, 1, 2, 2, 1, 2, 1, 1, 2, 1, 2, 2, 1, 2, 1, 1, 2) in which (1, 1) or (2, 2) means the trainer performs the training for 2 subsequent local epochs; and does only 1 local epoch in the case of (1, 2) or (2, 1).

The Adam optimizer is used with an initial learning rate of 10^{-5}, weight decay of 10^{-2} at each trainer, as in [14]. The learning rate is reduced on a plateau after 5 central epochs with a factor of 0.3. The trainers save and test every checkpoint of the model on the test dataset of Croatia. If the validation dataset of Croatia can be shared among the trainers, they can only save the checkpoint with the smallest validation loss to save disk space, if necessary. The AUC scores on the Croatia test set are given in Table 3. The scores demonstrate that our system outperforms previous results, which confirms the merits of greater quantities of data when using a deep learning approach. Additional experiments have also been done with Adam variants (AMSGrad [68], AdamX [69]), yielding similar AUC scores approximately 0.924 and all are superior to the previous best AUC score of 0.911 on the Croatia test set. The entire training time of our proposed system is less than 20 minutes, and the communication (including upload and download) of the encrypted weight from each distributed trainer with the server is approximately $234\ (\text{MB}) \times 20 \times 2 = 9.36\ (\text{GB})$.

Table 3. Area-under-the-curve (AUC) scores of learning methods on MRI datasets.

Paper	Method	AUC score
Stajduhar et al. [15]	Support Vector Machine	0.894
Bien et al. [14]	Neural Network	0.824
Bien et al. [14]	Neural Network	0.911
**This work (our system)**	Neural Network	0.924

https://doi.org/10.1371/journal.pone.0272423.t003

4.2 Experiment with ChestX-ray14 dataset

ChestX-ray14 dataset and its partition. The ChestX-ray14 dataset [13] contains 112,120 frontal-view chest X-ray images individually labeled with 14 different thoracic diseases: Atelectasis, Cardiomegaly, Effusion, Infiltration, Mass, Nodule, Pneumonia, Pneumothorax, Consolidation, Edema, Emphysema, Fibrosis, Pleural Thickening, Hernia. Following previous works [3, 13, 70, 71], this dataset is split into three partitions of training, validation, and test datasets with a ratio of 70:10:20 approximately. The number of images are 78468 (of 21528 patients), 11219 (of 3090 patients), 22433 (of 6187 patients) respectively in the datasets. There is no patient overlap between the sets.

Let us set the number of distributed trainers to $N = 5$, for concrete discussion. Trainer 0 has the ImageNet dataset. Other trainers (1, . . ., 4) possess approximately 78468/4 images from the training dataset described above.

Neural network models. Vertical trainer 0 trains DenseNet-121 [72] as the model M with the ImageNet dataset. In addition, horizontal trainers (1, . . ., 4) utilize the code given in [71] which also employs DenseNet-121 as the common neural network model M. However, the last layer of DenseNet-121 of 1000 neural nodes (for ImageNet) is replaced by 14 nodes equipped with sigmoid nonlinearity as described in Section 2, corresponding to the predicted probabilities of the 14 thoracic diseases listed above. These replacements in DenseNet-121 form the neural network models $M \circ M_1$, $M \circ M_2$, $M \circ M_3$, $M \circ M_4$ of the 4 distributed trainers. These models have 6,968,206 trainable parameters, of size 28 MB when saved to disk.

Authenticated encryption of weights. We use encrypt-then-mac method which is proved to be authenticated encryption [63], whose running time is less than 0.2 seconds when applied to a model weight of 28 MB. The ciphertext is also of 28 MB when saved to disk, and needs less than 1 second to be transmitted to the central parameter server. It is worth noting that the running time of encrypt-then-mac (0.2 seconds) and encrypted weight transmission (1 second) is negligible when compared with the time for training (feedforward and backpropagation on GPU) of approximately 60 seconds. Therefore, the overhead added by cryptographic operations and communications can be very small.

Early sharing for improved accuracy. Because each trainer has unbalanced classes, and the data are not independent and not identically distributed (non-iid), each trainer decides not to train on its entire local dataset but train on a part of the dataset before sending out the weight. This helps improve accuracy because the trained weight is expectedly not biased toward a particular local dataset. In particular, each trainer in our system uniformly at random splits its local data into 20 parts (each of which has approximately $78468/(4 \times 20) = 980$ images), trains the neural network on a partition each time and sends the weight out after one pass over that partition.

Loss function for training. The distributed Trainers 1, . . ., 4 use the same loss function of binary cross entropy. More precisely, for a single data item (X, y) in the training set, the loss function is defined as

$$L(X,y) = \sum_{c=1}^{14} [-y_c \cdot \log \Pr[Y_c = 1|X] - (1-y_c) \cdot \log \Pr[Y_c = 0|X]]$$

where $y = (y_1, \ldots, y_{14})$ is a label, $\Pr[Y_c = 1|X]$ is the predicted probability that the image contains pathology c given X, and $\Pr[Y_c = 0|X]$ is the predicted probability that the image does not contain pathology c given X.

Training details. Each trainer uses a batch size of 8 images, selects a random parti-tion of 980 images of its local data, and makes one pass (of feedforward and backpropagation) over that partition, which requires approximately 60 seconds. Each image is downscaled to a size of

Table 4. Area-under-the-curve (AUC) scores of learning methods on ChestX-ray14.

	Wang et al. [13]	Yao et al. [70]	Zech [71]	Our system	CheXNet [3]
Atelectasis	0.716	0.772	0.8161	0.8176	0.8094
Cardiomegaly	0.807	0.904	0.9105	0.9143	0.9248
Effusion	0.784	0.859	0.8839	0.8842	0.8638
Infiltration	0.609	0.695	0.7077	0.7098	0.7345
Mass	0.706	0.792	0.8308	0.8494	0.8676
Nodule	0.671	0.717	0.7748	0.7829	0.7802
Pneumonia	0.633	0.713	0.7651	0.7675	0.7680
Pneumothorax	0.806	0.841	0.8739	0.8762	0.8887
Consolidation	0.708	0.788	0.8008	0.8077	0.7901
Edema	0.835	0.882	0.8979	0.8931	0.8878
Emphysema	0.815	0.829	0.9227	0.9340	0.9371
Fibrosis	0.769	0.767	0.8293	0.8258	0.8047
Pleural Thickening	0.708	0.765	0.7860	0.7851	0.8062
Hernia	0.767	0.914	0.9010	0.9087	0.9164
**Average**	**0.7381**	**0.8027**	**0.8358**	**0.8397**	**0.8414**
Securely distributed training?	**no**	**no**	**no**	**yes**	**no**

https://doi.org/10.1371/journal.pone.0272423.t004

224×224, and normalized based on the mean ([0.485, 0.456, 0.406]) and standard deviation ([0.229, 0.224, 0.225]) of images in the ImageNet training set. Data augmentation is applied as follows: each image is horizontally flipped and randomly rotated by at most 45°. The stochastic gradient descent (SGD) optimizer is used with a momentum of 0.9, initial learning rate of 0.01, and weight decay of 10^{-4}. The number of central epochs is set to 15, which is the number of times passing through all (i.e. 78468) original training images. The learning rate lr in each central epoch is decreased by the following rule, for $0 \leq \mathsf{ce} \leq 14$,

$$\mathsf{lr} = \mathsf{lr} \times (0.5^{\lfloor \mathsf{ce}/2 \rfloor})$$

where $\lfloor \mathsf{ce}/2 \rfloor$ is the integer part of $\mathsf{ce}/2$.

Our system with the above distributed trainers produces an AUC score of 0.8397, which is smaller than that in [3] but larger than those in [13, 70, 71] as reported in Table 4. It should be noted that our AUC score is based on distributed training while the others are based only on centralized training. The total running time and ciphertext communication of our system are approximately 20 hours and 34 GB for 15 central epochs.

5 Conclusion

In this paper, we design a secure system for distributed learning with the following features: (1) distributed trainers can detect malicious activities in the server via authenticated encryption; (2) distributed trainers can perform both vertical and horizontal neural network training. We conduct experiments with datasets of MRI and X-ray images and obtain promising AUC scores for our proposed system when training with the datasets.

Author Contributions

Conceptualization: Le Trieu Phong.

Formal analysis: Le Trieu Phong.

Investigation: Le Trieu Phong.

Methodology: Le Trieu Phong.

Software: Le Trieu Phong.

Writing – original draft: Le Trieu Phong.

Writing – review & editing: Le Trieu Phong.

References

1. Esteva A, Kuprel B, Novoa RA, Ko J, Swetter SM, Blau HM, et al. Dermatologist-level classification of skin cancer with deep neural networks. Nature. 2017; 542:115–118. https://doi.org/10.1038/nature21056 PMID: 28117445
2. Gulshan V, Peng L, Coram M, Stumpe MC, Wu D, Narayanaswamy A, et al. Development and Validation of a Deep Learning Algorithm for Detection of Diabetic Retinopathy in Retinal Fundus Photographs. JAMA. 2016; 316(22):2402–2410. https://doi.org/10.1001/jama.2016.17216 PMID: 27898976
3. Rajpurkar P, Irvin J, Zhu K, Yang B, Mehta H, Duan T, et al. CheXNet: Radiologist-Level Pneumonia Detection on Chest X-Rays with Deep Learning. CoRR. 2017;abs/1711.05225.
4. Sarki R, Ahmed K, Wang H, Zhang Y, Wang K. Automated detection of COVID-19 through convolutional neural network using chest x-ray images. In: PLoS ONE 17(1): e0262052; 2022. Available from: https://doi.org/10.1371/journal.pone.0262052. https://doi.org/10.1371/journal.pone.0262052 PMID: 35061767
5. Stanford Medicine 2017 Health Trends Report: Harnessing the Power of Data in Health; 2017. https://med.stanford.edu/content/dam/sm/sm-news/documents/StanfordMedicineHealthTrendsWhitePaper2017.pdf.
6. Recht B, Ré C, Wright SJ, Niu F. Hogwild: A Lock-Free Approach to Parallelizing Stochastic Gradient Descent. In: NIPS 2011; 2011. p. 693–701. Available from: http://papers.nips.cc/paper/4390-hogwild-a-lock-free-approach-to-parallelizing-stochastic-gradient-descent.
7. Dean J, Corrado G, Monga R, Chen K, Devin M, Le QV, et al. Large Scale Distributed Deep Networks. In: 26th Annual Conference on Neural Information Processing Systems 2012.; 2012. p. 1232–1240. Available from: http://papers.nips.cc/paper/4687-large-scale-distributed-deep-networks.
8. Shokri R, Shmatikov V. Privacy-Preserving Deep Learning. In: Proceedings of the 22nd ACM SIGSAC Conference on Computer and Communications Security, 2015; 2015. p. 1310–1321. Available from: http://doi.acm.org/10.1145/2810103.2813687.
9. Phong LT, Aono Y, Hayashi T, Wang L, Moriai S. Privacy-Preserving Deep Learning via Additively Homomorphic Encryption. IEEE Trans Information Forensics and Security. 2018; 13(5):1333–1345. https://doi.org/10.1109/TIFS.2017.2787987
10. Phong LT, Phuong TT. Privacy-Preserving Deep Learning via Weight Transmission. IEEE Trans Information Forensics and Security. 2019; 14(11):3003–3015. https://doi.org/10.1109/TIFS.2019.2911169
11. Bonawitz K, Ivanov V, Kreuter B, Marcedone A, McMahan HB, Patel S, et al. Practical Secure Aggregation for Privacy-Preserving Machine Learning. In: Proceedings of the 2017 ACM SIGSAC Conference on Computer and Communications Security, CCS 2017; 2017. p. 1175–1191. Available from: http://doi.acm.org/10.1145/3133956.3133982.
12. McMahan B, Moore E, Ramage D, Hampson S, y Arcas BA. Communication-Efficient Learning of Deep Networks from Decentralized Data. In: Proceedings of the 20th International Conference on Artificial Intelligence and Statistics, AISTATS 2017; 2017. p. 1273–1282. Available from: http://proceedings.mlr.press/v54/mcmahan17a.html.
13. Wang X, Peng Y, Lu L, Lu Z, Bagheri M, Summers RM. ChestX-Ray8: Hospital-Scale Chest X-Ray Database and Benchmarks on Weakly-Supervised Classification and Localization of Common Thorax Diseases. In: 2017 IEEE Conference on Computer Vision and Pattern Recognition, CVPR 2017; 2017. p. 3462–3471. Available from: https://doi.org/10.1109/CVPR.2017.369.
14. Bien N, Rajpurkar P, Ball RL, Irvin J, Park A, Jones E, et al. MRNet: Deep-learning-assisted diagnosis for knee magnetic resonance imaging. PLoS Med 15(11): e1002699. 2018;. https://doi.org/10.1371/journal.pmed.1002699 PMID: 30481176
15. Stajduhar I, Mamula M, Miletic D, Ünal GB. Semi-automated detection of anterior cruciate ligament injury from MRI. Computer Methods and Programs in Biomedicine. 2017; 140:151–164. https://doi.org/10.1016/j.cmpb.2016.12.006 PMID: 28254071
16. Abadi M, Barham P, Chen J, Chen Z, Davis A, Dean J, et al. TensorFlow: A System for Large-Scale Machine Learning. In: Keeton K, Roscoe T, editors. 12th USENIX Symposium on Operating Systems Design and Implementation, OSDI 2016, Savannah, GA, USA, November 2-4, 2016. USENIX

Association; 2016. p. 265–283. Available from: https://www.usenix.org/conference/osdi16/technical-sessions/presentation/abadi.

17. Abadi M, Chu A, Goodfellow IJ, McMahan HB, Mironov I, Talwar K, et al. Deep Learning with Differential Privacy. In: Proceedings of the 2016 ACM SIGSAC Conference on Computer and Communications Security; 2016. p. 308–318. Available from: http://doi.acm.org/10.1145/2976749.2978318.
18. Jain P, Kulkarni V, Thakurta A, Williams O. To Drop or Not to Drop: Robustness, Consistency and Differential Privacy Properties of Dropout. CoRR. 2015;abs/1503.02031.
19. Agarwal N, Suresh AT, Yu FX, Kumar S, McMahan B. cpSGD: Communication-efficient and differentially-private distributed SGD. In: Advances in Neural Information Processing Systems 31: Annual Conference on Neural Information Processing Systems 2018, NeurIPS 2018, 3-8 December 2018, Montréal, Canada.; 2018. p. 7575–7586.
20. Yu L, Liu L, Pu C, Gursoy ME, Truex S. Differentially Private Model Publishing for Deep Learning. CoRR. 2019;abs/1904.02200.
21. Phuong TT, Phong LT. Distributed differentially-private learning with communication efficiency. Journal of Systems Architecture. 2022; p. 102555. https://doi.org/10.1016/j.sysarc.2022.102555
22. Danezis G, Diaz C. A Survey of Anonymous Communication Channels; 2008.
23. Nasr M, Shokri R, Houmansadr A. Machine Learning with Membership Privacy using Adversarial Regularization. In: Proceedings of the 2018 ACM SIGSAC Conference on Computer and Communications Security, CCS 2018; 2018. p. 634–646. Available from: https://doi.org/10.1145/3243734.3243855.
24. Dwork C, Naor M. On the difficulties of disclosure prevention in statistical databases or the case for differential privacy. J Privacy and Confidentiality. 2010; 2(1):93–107. https://doi.org/10.29012/jpc.v2i1.585
25. Fredrikson M, Lantz E, Jha S, Lin S, Page D, Ristenpart T. Privacy in Pharmacogenetics: An End-to-End Case Study of Personalized Warfarin Dosing. In: Proceedings of the 23rd USENIX Security Symposium, 2014.; 2014. p. 17–32. PMID: 27077138
26. Fredrikson M, Jha S, Ristenpart T. Model Inversion Attacks that Exploit Confidence Information and Basic Countermeasures. In: Proceedings of the 22nd ACM SIGSAC Conference on Computer and Communications Security, Denver, CO, USA, October 12-16, 2015; 2015. p. 1322–1333. Available from: https://doi.org/10.1145/2810103.2813677.
27. Shokri R, Stronati M, Song C, Shmatikov V. Membership Inference Attacks Against Machine Learning Models. In: 2017 IEEE Symposium on Security and Privacy, SP 2017, San Jose, CA, USA, May 22-26, 2017; 2017. p. 3–18. Available from: https://doi.org/10.1109/SP.2017.41.
28. Hitaj B, Ateniese G, Pérez-Cruz F. Deep Models Under the GAN: Information Leakage from Collaborative Deep Learning. In: Proceedings of the 2017 ACM SIGSAC Conference on Computer and Communications Security, CCS 2017; 2017. p. 603–618. Available from: http://doi.acm.org/10.1145/3133956.3134012.
29. Nasr M, Shokri R, Houmansadr A. Comprehensive Privacy Analysis of Deep Learning: Stand-alone and Federated Learning under Passive and Active White-box Inference Attacks. CoRR. 2018;abs/1812.00910.
30. Phong LT, Aono Y, Hayashi T, Wang L, Moriai S. Privacy-Preserving Deep Learning: Revisited and Enhanced. In: Applications and Techniques in Information Security—8th International Conference, ATIS 2017, Auckland, New Zealand, July 6-7, 2017, Proceedings; 2017. p. 100–110. Available from: https://doi.org/10.1007/978-981-10-5421-1_9.
31. Zhu L, Liu Z, Han S. Deep Leakage from Gradients. CoRR. 2019;abs/1906.08935.
32. Melis L, Song C, Cristofaro ED, Shmatikov V. Inference Attacks Against Collaborative Learning. CoRR. 2018;abs/1805.04049.
33. Nikolaenko V, Weinsberg U, Ioannidis S, Joye M, Boneh D, Taft N. Privacy-Preserving Ridge Regression on Hundreds of Millions of Records. In: 2013 IEEE Symposium on Security and Privacy, SP 2013, Berkeley, CA, USA, May 19-22, 2013; 2013. p. 334–348. Available from: https://doi.org/10.1109/SP.2013.30.
34. Zheng W, Popa RA, Gonzalez JE, Stoica I. Helen: Maliciously Secure Coopetitive Learning for Linear Models. CoRR. 2019;abs/1907.07212.
35. Hall R, Fienberg SE, Nardi Y. Secure Multiple Linear Regression Based on Homomorphic Encryption. Journal of Official Statistics. 2011;.
36. Gascón A, Schoppmann P, Balle B, Raykova M, Doerner J, Zahur S, et al. Privacy-Preserving Distributed Linear Regression on High-Dimensional Data. PoPETs. 2017; 2017(4):345–364.
37. Giacomelli I, Jha S, Joye M, Page CD, Yoon K. Privacy-Preserving Ridge Regression with only Linearly-Homomorphic Encryption. In: Applied Cryptography and Network Security—16th International Conference, ACNS 2018, Leuven, Belgium, July 2-4, 2018, Proceedings; 2018. p. 243–261. Available from: https://doi.org/10.1007/978-3-319-93387-0_13.

38. Cock MD, Dowsley R, Nascimento ACA, Newman SC. Fast, Privacy Preserving Linear Regression over Distributed Datasets based on Pre-Distributed Data. In: Proceedings of the 8th ACM Workshop on Artificial Intelligence and Security, AISec 2015, Denver, Colorado, USA, October 16, 2015; 2015. p. 3–14. Available from: https://doi.org/10.1145/2808769.2808774.

39. Aono Y, Hayashi T, Phong LT, Wang L. Input and Output Privacy-Preserving Linear Regression. IEICE Transactions. 2017; 100-D(10):2339–2347. https://doi.org/10.1587/transinf.2016INP0019

40. Aono Y, Hayashi T, Phong LT, Wang L. Privacy-Preserving Logistic Regression with Distributed Data Sources via Homomorphic Encryption. IEICE Transactions. 2016; 99-D(8):2079–2089. https://doi.org/10.1587/transinf.2015INP0020

41. Mohassel P, Zhang Y. SecureML: A System for Scalable Privacy-Preserving Machine Learning. In: 2017 IEEE Symposium on Security and Privacy; 2017. p. 19–38. Available from: https://doi.org/10.1109/SP.2017.12.

42. Mohassel P, Rindal P. ABY^3: A Mixed Protocol Framework for Machine Learning. In: Proceedings of the 2018 ACM SIGSAC Conference on Computer and Communications Security, CCS 2018; 2018. p. 35–52. Available from: https://doi.org/10.1145/3243734.3243760.

43. Gilad-Bachrach R, Dowlin N, Laine K, Lauter KE, Naehrig M, Wernsing J. CryptoNets: Applying Neural Networks to Encrypted Data with High Throughput and Accuracy. In: Proceedings of the 33rd International Conference on Machine Learning, ICML 2016; 2016. p. 201–210. Available from: http://jmlr.org/proceedings/papers/v48/gilad-bachrach16.html.

44. Liu J, Juuti M, Lu Y, Asokan N. Oblivious Neural Network Predictions via MiniONN Transformations. In: Proceedings of the 2017 ACM SIGSAC Conference on Computer and Communications Security. CCS'17. New York, NY, USA: ACM; 2017. p. 619–631. Available from: http://doi.acm.org/10.1145/3133956.3134056.

45. Riazi MS, Weinert C, Tkachenko O, Songhori EM, Schneider T, Koushanfar F. Chameleon: A Hybrid Secure Computation Framework for Machine Learning Applications. In: Proceedings of the 2018 on Asia Conference on Computer and Communications Security. ASIACCS'18. ACM; 2018. p. 707–721. Available from: http://doi.acm.org/10.1145/3196494.3196522.

46. Rouhani BD, Riazi MS, Koushanfar F. Deepsecure: Scalable Provably-secure Deep Learning. In: Proceedings of the 55th Annual Design Automation Conference. DAC'18. ACM; 2018. p. 2:1–2:6. Available from: http://doi.acm.org/10.1145/3195970.3196023.

47. Juvekar C, Vaikuntanathan V, Chandrakasan A. GAZELLE: A Low Latency Framework for Secure Neural Network Inference. In: 27th USENIX Security Symposium, USENIX Security 2018.; 2018. p. 1651–1669. Available from: https://www.usenix.org/conference/usenixsecurity18/presentation/juvekar.

48. Jiang X, Kim M, Lauter KE, Song Y. Secure Outsourced Matrix Computation and Application to Neural Networks. In: Proceedings of the 2018 ACM SIGSAC Conference on Computer and Communications Security, CCS 2018, Toronto, ON, Canada, October 15-19, 2018; 2018. p. 1209–1222. Available from: https://doi.org/10.1145/3243734.3243837.

49. Chang K, Balachandar N, Lam C, Yi D, Brown J, Beers A, et al. Distributed deep learning networks among institutions for medical imaging. Journal of the American Medical Informatics Association. 2018; 25:945–954. https://doi.org/10.1093/jamia/ocy017 PMID: 29617797

50. Gupta O, Raskar R. Distributed learning of deep neural network over multiple agents. J Network and Computer Applications. 2018; 116:1–8. https://doi.org/10.1016/j.comnet.2018.01.028

51. McClure P , Zheng CY , Kaczmarzyk J , Rogers-Lee J , Ghosh SS , Nielson D , et al. Distributed Weight Consolidation: A Brain Segmentation Case Study. In: Advances in Neural Information Processing Systems 31: Annual Conference on Neural Information Processing Systems 2018, NeurIPS 2018, 3-8 December 2018, Montréal, Canada.; 2018. p. 4097–4107. Available from: http://papers.nips.cc/paper/7664-distributed-weight-consolidation-a-brain-segmentation-case-study.

52. Ohrimenko O , Schuster F , Fournet C , Mehta A , Nowozin S , Vaswani K , et al. Oblivious Multi-party Machine Learning on Trusted Processors. In: Proceedings of the 25th USENIX Conference on Security Symposium. SEC'16. Berkeley, CA, USA: USENIX Association; 2016. p. 619–636. Available from: http://dl.acm.org/citation.cfm?id=3241094.3241143.

53. Kairouz P, McMahan HB, Avent B, Bellet A, Bennis M, Bhagoji AN, et al. Advances and Open Problems in Federated Learning. CoRR. 2019;abs/1912.04977.

54. Zheng S, Huang Z, Kwok JT. Communication-Efficient Distributed Blockwise Momentum SGD with Error-Feedback. In: Wallach HM, Larochelle H, Beygelzimer A, d'Alché-Buc F, Fox EB, Garnett R, editors. Advances in Neural Information Processing Systems 32: Annual Conference on Neural Information Processing Systems 2019, NeurIPS 2019, December 8-14, 2019, Vancouver, BC, Canada; 2019. p. 11446–11456. Available from: https://proceedings.neurips.cc/paper/2019/hash/80c0e8c4457441901351e4abbcf8c75c-Abstract.html.

55. Phuong TT, Phong LT. Communication-Efficient Distributed SGD with Error-Feedback, Revisited. Int J Comput Intell Syst. 2021; 14(1):1373–1387. https://doi.org/10.2991/ijcis.d.210412.001

56. Phong LT, Phuong TT. Distributed SignSGD With Improved Accuracy and Network-Fault Tolerance. IEEE Access. 2020; 8:191839–191849. https://doi.org/10.1109/ACCESS.2020.3032637

57. Phuong TT, Phong LT. Distributed SGD With Flexible Gradient Compression. IEEE Access. 2020; 8:64707–64717. https://doi.org/10.1109/ACCESS.2020.2984633

58. Cao T, Huu TT, Tran H, Tran K. A federated deep learning framework for privacy preservation and communication efficiency. J Syst Archit. 2022; 124:102413. https://doi.org/10.1016/j.sysarc.2022.102413

59. Ben-Or M, Goldwasser S, Wigderson A. Completeness Theorems for Non-Cryptographic Fault-Tolerant Distributed Computation (Extended Abstract). In: Simon J, editor. Proceedings of the 20th Annual ACM Symposium on Theory of Computing, May 2-4, 1988, Chicago, Illinois, USA. ACM; 1988. p. 1–10. Available from: https://doi.org/10.1145/62212.62213.

60. Damgård I, Pastro V, Smart NP, Zakarias S. Multiparty Computation from Somewhat Homomorphic Encryption. In: Safavi-Naini R, Canetti R, editors. Advances in Cryptology - CRYPTO 2012—32nd Annual Cryptology Conference, Santa Barbara, CA, USA, August 19-23, 2012. Proceedings. vol. 7417 of Lecture Notes in Computer Science. Springer; 2012. p. 643–662. Available from: https://doi.org/10.1007/978-3-642-32009-5_38.

61. Sotthiwat E, Zhen L, Li Z, Zhang C. Partially Encrypted Multi-Party Computation for Federated Learning. In: 2021 IEEE/ACM 21st International Symposium on Cluster, Cloud and Internet Computing (CCGrid); 2021. p. 828–835. https://doi.org/10.1109/CCGrid51090.2021.00101

62. Byrd D, Polychroniadou A. Differentially private secure multi-party computation for federated learning in financial applications. In: Balch T, editor. ICAIF'20: The First ACM International Conference on AI in Finance, New York, NY, USA, October 15-16, 2020. ACM; 2020. p. 16:1–16:9. Available from: https://doi.org/10.1145/3383455.3422562.

63. Bellare M, Namprempre C. Authenticated Encryption: Relations Among Notions and Analysis of the Generic Composition Paradigm. In: Proceedings of the 6th International Conference on the Theory and Application of Cryptology and Information Security: Advances in Cryptology. ASIACRYPT'00. Berlin, Heidelberg: Springer-Verlag; 2000. p. 531–545. Available from: http://dl.acm.org/citation.cfm?id=647096.716997.

64. Bellare M. New Proofs for NMAC and HMAC: Security without Collision Resistance. J Cryptology. 2015; 28(4):844–878. https://doi.org/10.1007/s00145-014-9185-x

65. Arpit D, Jastrzkebski S, Ballas N, Krueger D, Bengio E, Kanwal MS, et al. A Closer Look at Memorization in Deep Networks. In: Proceedings of the 34th International Conference on Machine Learning, ICML 2017; 2017. p. 233–242. Available from: http://proceedings.mlr.press/v70/arpit17a.html.

66. Hendrycks D, Lee K, Mazeika M. Using Pre-Training Can Improve Model Robustness and Uncertainty. In: Proceedings of the 36th International Conference on Machine Learning, ICML 2019, USA; 2019. p. 2712–2721. Available from: http://proceedings.mlr.press/v97/hendrycks19a.html.

67. Krizhevsky A, Sutskever I, Hinton GE. ImageNet Classification with Deep Convolutional Neural Networks. In: Advances in Neural Information Processing Systems 25: 26th Annual Conference on Neural Information Processing Systems 2012; 2012. p. 1106–1114. Available from: http://papers.nips.cc/paper/4824-imagenet-classification-with-deep-convolutional-neural-networks.

68. Reddi SJ, Kale S, Kumar S. On the Convergence of Adam and Beyond. In: International Conference on Learning Representations; 2018. Available from: https://openreview.net/forum?id=ryQu7f-RZ.

69. Phuong TT, Phong LT. On the Convergence Proof of AMSGrad and a New Version. IEEE Access. 2019; 7:61706–61716. https://doi.org/10.1109/ACCESS.2019.2916341

70. Yao L, Poblenz E, Dagunts D, Covington B, Bernard D, Lyman K. Learning to diagnose from scratch by exploiting dependencies among labels. CoRR. 2017;abs/1710.10501.

71. Zech J. reproduce-chexnet; 2018. https://github.com/jrzech/reproduce-chexnet.

72. Huang G, Liu Z, van der Maaten L, Weinberger KQ. Densely Connected Convolutional Networks. In: 2017 IEEE Conference on Computer Vision and Pattern Recognition, CVPR 2017; 2017. p. 2261–2269. Available from: https://doi.org/10.1109/CVPR.2017.243.
